# Caregiver quality of life and burden in rare genetic diseases in South Korea

**DOI:** 10.1097/MD.0000000000048006

**Published:** 2026-03-13

**Authors:** Sunyoung Choi, Ja Hye Kim, Gu-Hwan Kim, Beom Hee Lee, In Hee Choi

**Affiliations:** a Department of Genetic Counseling, University of Ulsan College of Medicine, Seoul, South Korea; b Department of Pediatrics, Asan Medical Center Children’s Hospital, University of Ulsan College of Medicine, Seoul, South Korea; c Medical Genetics Center, Asan Medical Center, University of Ulsan College of Medicine, Seoul, South Korea.

**Keywords:** caregiver burden, caregiver quality of life, caregiver support, genetic counseling, rare genetic diseases

## Abstract

Caregivers of individuals with rare genetic diseases experience substantial and persistent challenges that negatively affect their quality of life (QoL) and increase their burden. This study explored factors associated with caregiver QoL and burden in South Korea, focusing on patient characteristics, treatment availability, and genetic counseling experience. A cross-sectional survey was conducted with 159 caregivers of patients with rare genetic diseases at a tertiary general hospital. Caregiver QoL and burden were measured using the Caregiver QoL Scale and the Korean version of the Burden Assessment Scale. Demographic and clinical characteristics were also collected. Statistical analyses were performed using R software. Group differences were evaluated using Welch *t* tests, Wilcoxon rank-sum tests, and one-way analysis of variance with post hoc tests. Correlation analyses examined associations between QoL and caregiver burden. Caregiver QoL was significantly higher among those caring for minors, whereas caregiver burden was significantly higher among those caring for patients with registered disabilities. Treatment availability was associated with higher caregiver QoL and lower burden. Disease category also influenced outcomes: caregivers of patients with progressive conditions and localized impairments reported significantly lower QoL than those caring for patients with chronic conditions with effective treatment or symptomatic care or stable conditions with disabilities. Conversely, caregivers of patients with fatal diseases lacking effective treatment reported significantly higher burden than those caring for patients with chronic conditions with effective treatment. Caregiver QoL and burden were strongly and negatively correlated. Most caregivers (68.6%) had no prior genetic counseling experience, although those with counseling experience reported higher family openness scores, a QoL subdomain. Caregiver QoL and burden are closely linked to patient characteristics, treatment availability, and contextual caregiving demands. Expanding access to effective treatments, improving service accessibility, and integrating genetic counseling into caregiver support systems may improve the well-being of families affected by rare genetic diseases.

## 1. Introduction

Caring for a family member with a rare genetic disease can be overwhelming and difficult. Monica Coenraads, the mother of a child with Rett syndrome, describes the experience: “You don’t know what you’re up against. It is daunting, and it will consume your life.”^[1]^ These families, often referred to as “orphans of the healthcare system,” face persistent physical, emotional, and financial burdens due to delayed diagnoses and substantial demands associated with long-term disease management.

Rare genetic diseases affect >300 million individuals worldwide, including approximately 500,000 individuals in South Korea.^[2,3]^ Curative treatments are unavailable or severely limited for most rare genetic diseases. In South Korea, the Rare Disease Management Act was enacted in 2015 to alleviate caregiving burdens and associated costs.^[4]^ Currently, 1314 patients with rare genetic diseases are eligible for partial financial assistance through the Special Copayment Reduction Program.^[5]^ However, even where disease management options exist, limited access to specialized services often exacerbates family burdens. In particular, genetic counseling a vital psychosocial support service, remains largely uncovered by the National Health Insurance due to the absence of established reimbursement. Consequently, access disparities persist, driven by the concentration of services in tertiary hospitals.^[6,7]^

Caregiver quality of life (QoL) is strongly influenced by caregiver burden.^[8]^ Excessive caregiving responsibilities can lead to stress, anxiety, chronic fatigue, and financial strain.^[8,9]^ The progressive and hereditary nature of many rare genetic diseases adds further psychological stressors, such as uncertainty about disease progression and concern regarding familial risk, further compromising the well-being of caregivers.^[10–12]^

Genetic counseling provides essential medical and genetic information and psychological support to help families understand and adapt to genetic conditions.^[13]^ Genetic counseling can reduce emotional stress, enhance disease understanding, and aid in informed decision-making.^[14–17]^ Nevertheless, caregivers of individuals with rare genetic diseases often encounter significant barriers to accessing essential healthcare services, which can negatively affect their QoL.^[18]^ A national survey revealed that 78.9% of Korean patients with rare genetic diseases and their families have never received genetic counseling,^[10]^ highlighting substantial unmet needs in the healthcare system. As of September 2024, the Korean Society of Medical Genetics and Genomics has issued 76 certified genetic counselors in Korea.^[19]^ However, because this certification lacks national recognition, employment opportunities and the scope of practice remain constrained. These systemic limitations contribute to a shortage of qualified professionals in medical genetics centers, thereby limiting equitable access to specialized counseling services for affected families.

Despite the acknowledged importance of psychosocial support, empirical evidence on the associations between caregiver burden, QoL, and access to services such as genetic counseling remains limited in South Korea. Therefore, this study aims to analyze factors associated with caregiver burden and QoL among caregivers of individuals with rare genetic diseases in South Korea. The results of this study will offer foundational evidence to strengthen caregiver-centered support systems and promote the integration of genetic counseling services into the national healthcare framework.

## 2. Methods

### 2.1. Study design

This cross-sectional quantitative survey investigated caregiver burden and QoL among individuals caring for patients with rare genetic diseases. The study was approved by the Institutional Review Board of Asan Medical Center (IRB number: 2023‐0211).

### 2.2. Participants

Caregivers of patients with confirmed rare genetic diseases were recruited at the Medical Genetics Center at Asan Medical Center, Seoul, South Korea, between February and June 2023 through convenience sampling. Eligibility criteria were: being the caregiver of a patient diagnosed with a rare genetic disease; and being aged 18 years or older, able to communicate, understand the questionnaire, and respond without cognitive impairments. Caregivers who declined participation or could not complete the survey independently were excluded. Of 160 participants who completed the survey, one was excluded for incomplete responses, yielding a final sample of 159.

### 2.3. Measures

#### 2.3.1. QoL scale

QoL was assessed using the Korean QoL scale developed for caregivers of individuals with developmental disabilities.^[20]^ It comprises 22 items across 5 domains: family psychological health (5 items), family burden (5 items), community participation and support (8 items), family openness (2 items), and family cohesion (2 items). Items are rated on a 5-point Likert scale, with higher scores indicating better QoL; 5 items are reverse-coded. Total scores range from 22 to 110. Internal consistency was high (Cronbach α = .928).

#### 2.3.2. Korean version of the Burden Assessment Scale

The Korean version of the Burden Assessment Scale, adapted from the original Burden Assessment Scale^[21]^ by Kwak et al,^[22]^ comprises 19 items across 3 domains: activity limitation (8 items), social strain (7 items), and feeling of worry and guilt (4 items). Items are rated on a 4-point Likert scale, with higher scores reflecting greater burden. Total scores range from 19 to 76, with high internal consistency reported in Korean samples (Cronbach’s α = .910).

#### 2.3.3. Demographic and clinical characteristics

A structured questionnaire collected sociodemographic data from caregivers (gender, age, relationship to the patient, marital status, education, employment, religion, monthly household income, and residential area) and patients’ clinical characteristics (gender, age group, diagnosis, inheritance pattern, family history, time since diagnosis, disability registration status, and experience with genetic counseling). Treatment availability was defined as the presence of disease-specific therapies that had received formal regulatory approval in South Korea (Ministry of Food and Drug Safety) or by the U.S. Food and Drug Administration and were clinically available during the study period. This definition includes disease-modifying or guideline-based management therapies but excludes supportive care that is limited to symptomatic or palliative purposes.

The disease categories were based on the classification system of the Ministry of Health and Welfare,^[23]^ which classifies rare diseases into 4 groups: chronic conditions with effective treatment, fatal diseases without treatment, symptomatic care for stable disabilities, and progressive localized impairments.

### 2.4. Procedures

Eligible participants received a detailed explanation of the study objectives and procedures. Written informed consent was obtained prior to enrollment. Self-administered questionnaires were distributed and collected by trained research staff. The responses were anonymized to ensure confidentiality. Permission to use and adapt the assessment tools was obtained from the original developers.

### 2.5. Statistical analysis

Analyses were performed using R software (version 2024.04.02; R Foundation for Statistical Computing, Vienna, Austria). Descriptive statistics were used to summarize the characteristics of the caregiver and patient. Group differences in caregiver QoL and caregiver burden were examined using Welch two-sample *t* tests (patient age group, treatment availability) and Wilcoxon rank-sum tests (disability registration status) depending on variable distribution. Differences across disease categories were assessed using one-way analysis of variance, with Tukey’s honestly significant difference tests for post hoc comparisons.

Pearson correlation analysis assessed the association between caregiver QoL and caregiver burden. All statistical tests were two-sided with significance set at *P* < .05. The test selection was based on data distribution and measurement scale.

## 3. Results

### 3.1. Characteristics of caregivers of rare genetic disease patients

Table 1 summarizes the demographic characteristics of the 159 caregivers included in the study. Most participants were female (67.9%) and aged 30 to 49 years (79.9%). The majority were parents of the patients (89.3%), followed by spouses (5.7%), children (4.4%), and siblings (0.6%). Geographic distribution was nearly even between metropolitan areas (49.6%) and smaller cities or rural areas (50.4%). Regarding education, 45.9% held a university degree, and 66.1% were employed. More than half (53.4%) reported a religious affiliation, and most were married (91.2%). Regarding income, 37.7% had an average monthly household income exceeding 5 million South Korean won.

**Table 1:** Caregiver characteristics of patients with rare genetic diseases (N = 159).

Characteristics	N (%)
Gender	
Female	108 (67.9)
Male	51 (32.1)
Age	
20–29 yr	2 (1.3)
30–39 yr	55 (34.6)
40–49 yr	72 (45.3)
50–59 yr	26 (16.4)
≥60	4 (2.5)
Relationship to the patient	
Parent	145 (89.3)
Other (e.g., spouse, child, sibling, grandparent)	14 (10.7)
Residential area	
Metropolitan area	79 (49.6)
Small city or rural area	80 (50.4)
Education level	
Middle school graduate	7 (4.4)
High school graduate	34 (21.4)
College graduate	34 (21.4)
University graduate	73 (45.9)
Graduate school or higher	11 (6.9)
Employment status	
Employed	105 (66.1)
Unemployed	54 (33.9)
Religion	
Christianity	36 (22.6)
Catholicism	23 (14.5)
Buddhism	26 (16.4)
None	72 (45.3)
Other	2 (1.3)
Marital status	
Single	1 (0.6)
Married	145 (91.2)
Divorced/separated	8 (5.0)
Widowed	3 (1.9)
Cohabiting/common-law	2 (1.3)
Monthly household income	
<1 million KRW	2 (1.3)
1–2 million KRW	6 (3.8)
2–3 million KRW	16 (10.1)
3–4 million KRW	18 (11.3)
4–5 million KRW	57 (35.8)
Over 5 million KRW	60 (37.7)

KRW = South Korean won.

### 3.2. Characteristics of rare genetic disease patients

As shown in Table 2, 76.7% of the patients with rare genetic diseases cared for by the participating caregivers were under 19 years of age, and 22.6% were registered as having a disability. Only 28.3% had effective treatment available, while 56.0% were classified as having chronic or stable conditions with disabilities. Autosomal dominant inheritance was the most common (44.0%), and 13.8% reported a family history of genetic disease. The time since diagnosis varied: 64.2% diagnosed within the past 5 years, 18.9% within 5 to 10 years, 8.2% within 10 to 15 years, and 8.8% for ≥15 years.

**Table 2:** Characteristics of patients with rare genetic diseases (N = 159).

Characteristics	N (%)
Gender	
Male	78 (49.1)
Female	81 (50.9)
Age	
<19 yr	122 (76.7)
≥19 yr	37 (23.3)
Disease category	
Chronic, treatment available (Group 1)	49 (30.8)
Fatal, no effective treatment (Group 2)	11 (6.9)
Symptomatic care or stable with disabilities (Group 3)	89 (56.0)
Progressive, localized impairments (Group 4)	10 (6.3)
Inheritance patterns	
Autosomal dominant	70 (44.0)
Autosomal recessive	33 (20.8)
X-linked	13 (8.2)
Mitochondrial	3 (1.9)
Other	40 (25.2)
Family history of genetic disease	
Yes	22 (13.8)
No	137 (86.2)
Time since diagnosis	
<5 yr	102 (64.2)
5–10 yr	30 (18.9)
10–15 yr	13 (8.2)
≥15 yr	14 (8.8)
Disability registration	
Registered	36 (22.6)
Not registered	123 (77.4)
Treatment availability	
Available	45 (28.3)
Not available	114 (71.7)
Copayment reduction registration	
Yes	116 (73.0)
No	43 (27.0)

### 3.3. Overall caregiver QoL and burden

The mean total QoL score was 83.25 ± 12.81 (range: 22–110), indicating a moderate level of QoL among caregivers. The mean caregiver burden score was 39.74 ± 11.78 (range: 19–76), reflecting moderate burden. Subdomain scores for QoL and caregiver burden are presented in Table 3.

**Table 3:** Caregiver quality of life and burden scores (N = 159).

Measure	Subdomain	Mean ± SD
Caregiver QoL	Total QoL score	83.25 ± 12.81
	Family psychological health	21.02 ± 3.08
	Family burden	16.46 ± 3.56
	Community participation and support	29.81 ± 6.22
	Family openness	7.93 ± 1.60
	Family cohesion	8.04 ± 1.63
Caregiver burden	Total burden score	39.74 ± 11.78
	Activity limitation	18.27 ± 6.56
	Social strain	11.80 ± 3.98
	Feelings of worry and guilt	9.67 ± 3.05

QoL = quality of life.

### 3.4. Caregiver QoL and burden by caregiver characteristics

Caregiver QoL and burden did not significantly differ according to caregivers’ demographic characteristics, including gender, age, residential area, education level, employment status, religion, marital status, and household income (Table S1, Supplemental Digital Content, https://links.lww.com/MD/R520). However, parent caregivers reported a significantly higher QoL than nonparent caregivers (84.82 ± 12.74 vs 77.94 ± 13.63; *P* = .038), whereas caregiver burden showed no significant difference by relationship to the patient (*P* = .96).

### 3.5. Caregiver QoL and burden by patient characteristics

The differences in caregiver QoL and burden according to patient characteristics are summarized in Table S2, Supplemental Digital Content, https://links.lww.com/MD/R520. Significant differences in caregiver outcomes were observed according to patient age groups, disability registration status, and treatment availability. Conversely, patient gender, inheritance pattern, family history of genetic disease, time since diagnosis and disease classification based on the Korean classification of diseases were not significantly associated with caregiver QoL or burden (all *P* > .05).

With respect to patient age, caregivers of minors reported significantly higher QoL than those caring for adults (85.48 ± 12.59 vs 79.49 ± 13.32; *P* = .013).

Regarding disability status, caregiver burden was significantly higher among those caring for patients with registered disabilities than among those without (44.50 ± 12.14 vs 38.24 ± 11.23, *P* = .004).

**Figure 1.**
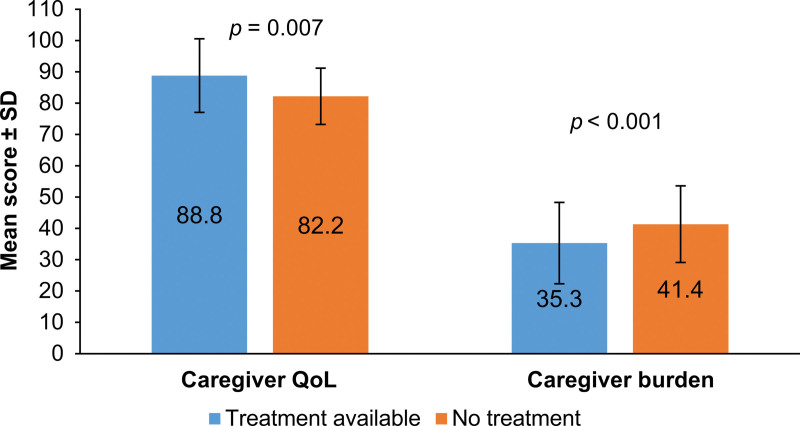
Impact of treatment availability on caregiver QoL and burden. Mean caregiver QoL and caregiver burden scores among independent caregivers, stratified by treatment availability. Error bars represent standard deviations. Statistically significant differences were observed between groups (*P* = .0026 for QoL; *P* < .001 for burden). QoL = quality of life.

Treatment availability was significantly associated with caregiver QoL and caregiver burden (Fig. 1). Caregivers of patients with treatment availability reported significantly higher QoL (88.82 ± 11.75) than those without (82.21 ± 13.00; *P* = .007). Similarly, caregiver burden was significantly lower when treatment was available (35.33 ± 8.99 vs 41.37 ± 12.23; *P* < .001).

Although disease classification based on the Korean classification of diseases was not significantly associated with caregiver outcomes, analyses using 4 clinically defined disease categories revealed significant differences in caregiver QoL and burden. One-way analysis of variance showed that disease category had a significant effect on both caregiver QoL (*F*[3155] = 4.42, *P* = .005) and caregiver burden (*F*[3155] = 3.32, *P* = .021). Tukey’s honestly significant difference test showed that caregivers in Group 4 reported significantly lower QoL than those in Group 1 (mean difference = −15.23, *P* = .003) and Group 3 (mean difference = −11.42, *P* = .036). No significant QoL differences were observed for Group 2 compared to Groups 1 (*P* = .392) or 3 (*P* = .896), or for Group 3 compared to Group 1 (*P* = .324).

For caregiver burden, caregivers of patients in Group 2 reported significantly higher scores than those in Group 1 (mean difference = 10.20, *P* = .041). All other group comparisons for burden were not statistically significant (all *P* > .05). These results suggest that burden was uniquely elevated in Group 2, while burden levels in Groups 1, 3, and 4 were statistically comparable.

**Figure 2.**
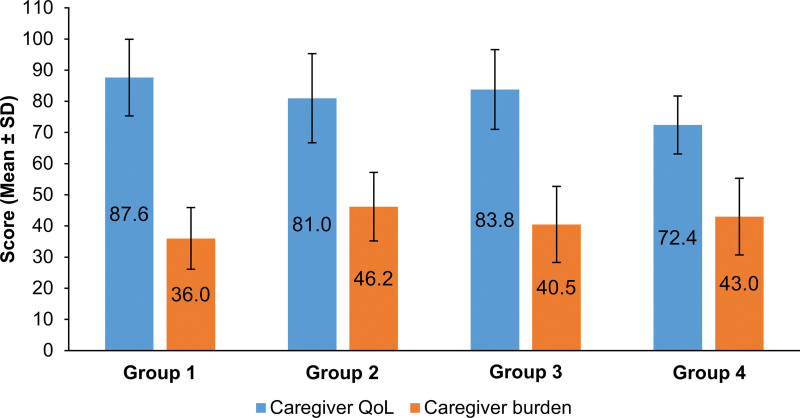
Comparison of caregiver QoL and burden across disease categories. Mean (±SD) caregiver QoL scores and caregiver burden by disease category scores. Group 1 = chronic conditions with effective treatment; Group 2 = fatal diseases with no effective treatment; Group 3 = symptomatic care or stable conditions with disabilities; Group 4 = progressive conditions with localized impairments. QoL = quality of life.

Figure 2 shows mean scores and standard deviations for caregiver QoL and burden across the 4 disease categories. These results indicate that QoL was lowest among caregivers in Group 4, while burden was highest in Group 2.

### 3.6. Correlation between caregiver QoL and caregiver burden

Pearson correlation analysis revealed a significant negative correlation between caregiver QoL and caregiver burden (*r* = −.668, *t*(157) = −11.26, *P* < .001; 95% CI: −.746 to −.572). This indicates that higher caregiver burden was associated with lower caregiver QoL.

### 3.7. Caregiver QoL and caregiver burden by genetic counseling experience

**Figure 3.**
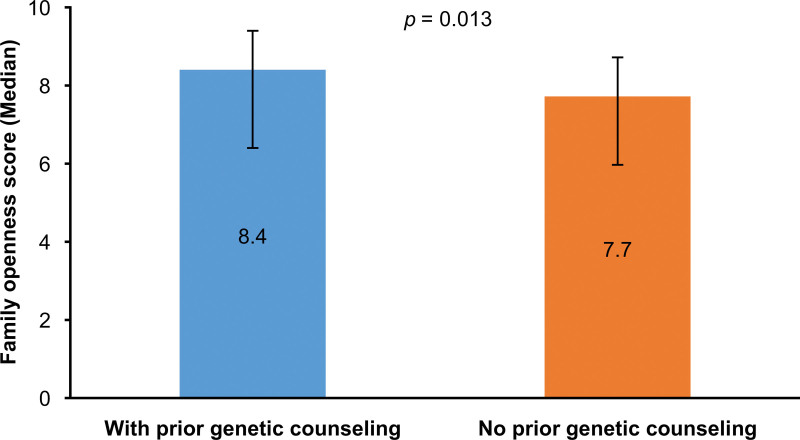
Family openness scores by genetic counseling experience. Median (IQR) scores on the family openness domain of the QoL scale are shown for caregivers with (n = 50) and without (n = 109) prior genetic counseling. Caregivers who had received genetic counseling reported significantly higher openness scores (median [IQR]: 9.0 [7.25–10.0] vs 8.0 [6.0–9.0]; *P* = .011, Wilcoxon rank-sum test). Error bars represent interquartile ranges. IQR = interquartile range, QoL = quality of life.

Of the 159 caregivers, 109 (68.6%) reported no prior experience of genetic counseling. Caregivers with prior counseling experience showed significantly higher scores on the family openness domain of QoL compared with those without counseling experience (8.40 ± 1.58 vs 7.72 ± 1.58, *P* = .013). However, we found no significant differences between the 2 groups in overall QoL (no experience: 83.80 ± 13.23; experience: 84.70 ± 12.48), the other 4 QoL subdomains, or caregiver burden (all *P* > .05) (Table S3, Supplemental Digital Content, https://links.lww.com/MD/R520). Figure 3 presents the distribution of openness scores by genetic counseling experience.

## 4. Discussion

This study examined significant factors influencing caregiver QoL and caregiver burden among families of patients with rare genetic diseases in South Korea. Caregivers of adult patients reported significantly lower QoL than those caring for minors, suggesting that the prolonged management of chronic conditions into adulthood may increase physical, psychological, and social caregiving demands. These findings align with prior studies showing that caregivers of older patients or individuals with disabilities experience heightened stress related to long-term care responsibilities, uncertainty about the future, reduced social participation, and financial challenges.^[12,24–28]^

Disability registration status was a significant determinant of caregiver burden. Caregivers of patients with registered disabilities reported a significantly higher burden, likely due to the elevated intensity of care and the need to manage rehabilitation, behavioral concerns, and navigation of social welfare systems. This finding aligns with prior research indicating that caregivers, particularly mothers of children with special needs, experience heightened stress as caregiving demands intensify.^[29]^ Regarding rare genetic diseases, disability registration often entails additional administrative, emotional, and logistical challenges, further compounding the burden.^[9,11,12,30]^ Such cumulative demands can negatively impact physical and mental health, reducing QoL and underscoring the need for targeted psychosocial support and improved accessibility to disability-related services.

Treatment availability also significantly influenced caregiver outcomes. Caregivers of patients with available treatment options reported higher QoL and lower burden than those without such options. This suggests that access to effective therapy may alleviate emotional distress, uncertainty, and feelings of helplessness, even when the care burden remains substantial. However, effective treatments are available for fewer than 5% of rare diseases, which contributes to delayed diagnoses, prognostic uncertainty, and heightened caregiver anxiety.^[12,31–34]^ Expanding therapeutic access and timely dissemination of disease-specific information, along with comprehensive psychosocial support, are therefore critical to improving caregiver well-being and reducing burden.

Disease category was associated with significant differences in both QoL and burden. Caregivers of patients with progressive conditions and localized impairments reported lower QoL, reflecting the psychological distress associated with anticipated decline, instability of disease trajectory, and high care demands.^[35–38]^ Persistent anxiety regarding disease progression, financial strain, and uncertainty may intensify psychosocial distress.^[9,11]^ In contrast, caregivers of patients with fatal diseases who lack treatment options reported a significantly higher burden than those caring for patients with chronic or stable conditions. This heightened burden may stem from the emotional strain associated with anticipatory grief and the challenges of managing life-limiting and unpredictable disease trajectories.^[12,36,37]^ Conversely, caregivers of patients with treatable or stable conditions tended to report more favorable QoL and burden outcomes, suggesting that the availability of effective therapies and more predictable care demands may buffer caregiver stress and promote emotional resilience.^[9,11]^

Collectively, these findings emphasize the need for tailored, diagnosis-specific psychosocial interventions and coordinated care-planning support for families affected by progressive or untreatable rare diseases.

A strong negative correlation between caregiver QoL and burden indicates that these constructs are closely interrelated, with higher burden directly undermining caregivers’ emotional and physical well-being. Similar associations have been observed among caregivers of patients with cancer and other chronic illnesses,^[15,39,40]^ highlighting the need for integrated support systems that simultaneously reduce burden and enhance QoL.

Genetic counseling experience was associated with significantly higher levels of family openness, a domain of QoL, reflecting improved communication and emotional adaptation within families. This supports earlier findings that genetic counseling can enhance coping, reduce distress, and improve psychosocial adjustment.^[14–17]^ Despite its benefits, most caregivers in this study had limited or no prior exposure to genetic counseling.

However, 68.6% of the caregivers in this study had never received genetic counseling, reflecting the limited use of these services in South Korea. Although a certification system for genetic counselors has been implemented by the Korean Society of Medical Genetics and Genomics, the profession lacks formal recognition within the national health insurance framework, thereby limiting accessibility.^[6,19]^ These structural constraints likely contribute to the low uptake of genetic counseling among families affected by rare genetic diseases and may partially account for the modest association between counseling experience and caregiver outcomes observed in this study. This underscores the need for enhanced accessibility and formal integration of genetic counseling services into the healthcare system.

Addressing these structural barriers will require the implementation of formal certification standards for genetic counselors, expanded insurance coverage, and system-level strategies to improve accessibility and service quality. Public awareness campaigns and the integration of counseling into routine medical practice could enhance utilization. Given the limited treatment options available for many rare genetic diseases, improving access to genetic counseling is likely to provide meaningful psychosocial support, reduce caregiver burden, and improve overall caregiver well-being.

This study has several limitations that should be considered when interpreting the findings. First, recruitment through purposive sampling from a single tertiary hospital limits generalizability, as caregiving experiences may differ across clinical and regional contexts. Additionally, the predominance of parent caregivers in the sample may further constrain generalizability to caregivers with other familial roles, given that caregiving responsibilities and perceived burden potentially vary according to the caregiver–patient relationship. Future multicenter studies with larger and more diverse samples are warranted to enhance representativeness. Second, the use of self-reported survey data may introduce recall or social desirability bias. Incorporating qualitative or mixed-method approaches could provide a more comprehensive understanding of caregivers’ experiences. Third, the cross-sectional design precludes inferences about causality or changes in caregiver QoL and burden over time. Longitudinal designs are needed to examine how caregiver outcomes evolve as disease trajectories progress and caregivers adapt to ongoing demands. Fourth, although the multivariable analyses included treatment availability, genetic counseling experience, disease category, caregiver and patient age, and time since diagnosis, the possibility of residual confounding cannot be ruled out. Disease category and time since diagnosis served as indirect indicators of clinical severity, but detailed information on functional impairment or symptom progression was not available. Future studies should incorporate objective measures of disease severity to clarify the independent influence of clinical characteristics and caregiving factors. Finally, although this study suggests that genetic counseling may benefit caregiver well-being, the limited availability and low utilization of such services in South Korea restrict broader applicability. More detailed indicators of caregiving demands, including the amount of direct care provided, challenges encountered when navigating medical and welfare systems, and the use of respite services, would also enhance the understanding of how service accessibility and caregiving responsibilities influence caregiver outcomes.

## 5. Conclusions

In conclusion, we found a positive correlation between treatment availability and caregiver QoL; however, no independent association was observed between treatment availability and caregiver burden. Other factors, including patient age, disability registration status, and disease type, further contributed to variations in caregiver experiences, highlighting the multifactorial nature of caregiver outcomes. The differences we observed in burden and QoL across disease categories and caregiver characteristics indicate that the experience of caregiving varies according to the trajectory of the disease and the stage of caregiving. Moreover, genetic counseling demonstrated domain-specific benefits to caregivers. It was found to improve family communication but was not associated with overall QoL or burden. Expanding access to effective treatments, developing age-tailored and stage-sensitive support services, and strengthening the integration of genetic counseling into rare disease care systems may improve caregiver outcomes.

## Acknowledgments

The researchers deeply appreciate the participation of the caregivers in this study.

## Author contributions

**Conceptualization:** Sunyoung Choi, In Hee Choi.

**Data curation:** Sunyoung Choi.

**Formal analysis:** Sunyoung Choi.

**Investigation:** Sunyoung Choi.

**Methodology:** Sunyoung Choi, In Hee Choi.

**Project administration:** Sunyoung Choi, In Hee Choi.

**Resources:** Ja Hye Kim, Beom Hee Lee.

**Supervision:** Beom Hee Lee, In Hee Choi.

**Validation:** Sunyoung Choi, In Hee Choi.

**Visualization:** Sunyoung Choi.

**Writing – original draft:** Sunyoung Choi, In Hee Choi.

**Writing – review & editing:** Ja Hye Kim, Gu-Hwan Kim, Beom Hee Lee, In Hee Choi.

## Supplementary Material
