# Supplementary material for: Caregiver quality of life and burden in rare genetic diseases in South Korea
Source: Medicine (Baltimore). 2026 Mar 13;105(11):e48006. doi: 10.1097/MD.0000000000048006 (PMC12991492; doi:10.1097/MD.0000000000048006)
Supplement: Supplementary file 1 [file medi-105-e48006-s001.docx]

**Supplementary Table S1.** Caregiver quality of life and burden by characteristics (N = 159)

| **Characteristic** | **N** | **QoL**  **(Mean ± SD)** | ***P*-value** | **Burden**  **(Mean ± SD)** | ***P*-value** |
| --- | --- | --- | --- | --- | --- |
| **Gender** |  |  | 0.29 |  | 0.46 |
| Male | 51 | 85.70 ± 12.39 |  | 38.64 ± 10.97 |  |
| Female | 108 | 83.34 ± 13.21 |  | 40.13 ± 12.05 |  |
| **Age group** |  |  | 0.40 |  | 0.52 |
| 20–29 years | 2 | 82.00 ± 9.90 |  | 37.50 ± 2.12 |  |
| 30–39 years | 55 | 86.10 ± 13.58 |  | 39.62 ± 12.17 |  |
| 40–49 years | 72 | 84.01 ± 12.08 |  | 38.54 ± 10.84 |  |
| 50–59 years | 26 | 81.90 ± 14.69 |  | 41.62 ± 13.36 |  |
| ≥60 years | 4 | 76.00 ± 4.08 |  | 47.25 ± 9.95 |  |
| **Relationship to the patient** |  |  | **0.038** |  | 0.96 |
| Parent | 145 | 84.82 ± 12.74 |  | 39.67 ± 11.54 |  |
| Other caregivers | 14 | 77.94 ± 13.63 |  | 39.59 ± 13.37 |  |
| **Residential area** |  |  | 0.66 |  | 0.38 |
| Metropolitan area or larger | 79 | 83.62 ± 11.89 |  | 38.83 ± 11.61 |  |
| Small or medium-sized city or rural area | 80 | 84.53 ± 13.99 |  | 40.46 ± 11.80 |  |
| **Education level** |  |  | 0.75 |  | 0.99 |
| Middle school graduate | 7 | 88.75 ± 11.90 |  | 39.50 ± 12.56 |  |
| High school graduate | 34 | 82.53 ± 15.28 |  | 39.97 ± 11.92 |  |
| College graduate | 34 | 84.94 ± 14.45 |  | 39.00 ± 12.47 |  |
| University graduate | 73 | 83.66 ± 11.85 |  | 39.70 ± 11.64 |  |
| Graduate school or higher | 11 | 85.73 ± 8.68 |  | 40.55 ± 10.44 |  |
| **Employment status** |  |  | 0.18 |  | 0.74 |
| Unemployed | 54 | 82.13 ± 13.16 |  | 40.25 ± 12.23 |  |
| Employed | 105 | 85.06 ± 12.82 |  | 39.37 ± 11.48 |  |
| **Religion** |  |  | 0.50 |  | 0.33 |
| Protestant | 36 | 82.34 ± 12.04 |  | 40.91 ± 11.41 |  |
| Catholic | 23 | 85.38 ± 13.32 |  | 40.83 ± 11.77 |  |
| Buddhist | 26 | 81.77 ± 15.84 |  | 42.08 ± 14.61 |  |
| None | 72 | 85.07 ± 12.30 |  | 37.58 ± 10.63 |  |
| Other | 2 | 93.50 ± 3.54 |  | 47.00 ± 1.41 |  |
| **Marital status** |  |  | 0.58 |  | 0.34 |
| Single (never married) | 1 | 78.00 ± NA |  | 45.00 ± NA |  |
| Married | 145 | 84.57 ± 12.86 |  | 39.12 ± 11.65 |  |
| Divorced/separated | 8 | 75.12 ± 15.53 |  | 46.75 ± 11.63 |  |
| Widowed | 3 | 86.00 ± 12.77 |  | 45.67 ± 8.02 |  |
| Cohabiting/in a common-law marriage | 2 | 85.00 ± 1.41 |  | 38.50 ± 20.51 |  |
| **Monthly household income** |  |  | 0.78 |  | 0.53 |
| <1 million KRW | 2 | 85.00 ± 1.41 |  | 38.50 ± 20.51 |  |
| 1–2 million KRW | 6 | 84.14 ± 12.65 |  | 41.57 ± 12.71 |  |
| 2–3 million KRW | 16 | 82.12 ± 16.50 |  | 43.44 ± 12.71 |  |
| 3–4 million KRW | 18 | 80.52 ± 12.56 |  | 42.00 ± 9.15 |  |
| 4–5 million KRW | 57 | 84.09 ± 13.29 |  | 38.57 ± 12.19 |  |
| Other | 60 | 85.80 ± 12.13 |  | 38.62 ± 11.59 |  |

*Note.* Statistically significant p values (*p* < 0.05) are shown in bold. KRW, South Korean won; QoL, quality of life.

**Supplementary Table S2.** Caregiver quality of life and burden by patient characteristics (N = 159)

| **Characteristic** | **N** | **QoL**  **(Mean ± SD)** | ***P*-value** | **Burden**  **(Mean ± SD)** | ***P*-value** |
| --- | --- | --- | --- | --- | --- |
| **Gender** |  |  | .84 |  | .60 |
| Male | 78 | 83.87 ± 13.83 |  | 40.15 ± 11.39 |  |
| Female | 81 | 84.28 ± 12.16 |  | 39.19 ± 12.04 |  |
| **Age group** |  |  | **.013** |  | .19 |
| <19 years | 122 | 85.48 ± 12.59 |  | 38.97 ± 11.16 |  |
| ≥19 years | 37 | 79.49 ± 13.32 |  | 41.95 ± 13.26 |  |
| **Disease classification (KCD)** |  |  | .34 |  | .09 |
| Neoplasms (C00–D48) | 4 | 84.50 ± 12.56 |  | 28.50 ± 8.43 |  |
| Diseases of the blood, hematopoietic, & immune systems (D50–D89) | 1 | 78.00 ± NA |  | 45.00 ± NA |  |
| Endocrine, nutritional & metabolic (E00–E90) | 37 | 86.59 ± 12.98 |  | 37.62 ± 12.01 |  |
| Nervous system (G00–G99) | 8 | 71.12 ± 11.36 |  | 49.75 ± 11.91 |  |
| Eye (H00–H59) | 3 | 81.00 ± 2.65 |  | 29.00 ± 10.54 |  |
| Circulatory (I00–I99) | 2 | 86.00 ± 18.38 |  | 36.50 ± 19.09 |  |
| Musculoskeletal (M00–M99) | 1 | 89.00 ± NA |  | 36.00 ± NA |  |
| Genito-urinary (N00–N99) | 1 | 84.00± NA |  | 45.00 ± NA |  |
| Congenital heart defects (Q20–Q28) | 2 | 75.50 ± 0.71 |  | 52.50 ± 2.12 |  |
| Other congenital malformations (Q80–Q89) | 77 | 83.83 ± 12.95 |  | 40.04 ± 11.38 |  |
| Chromosomal abnormalities (Q90–Q99) | 23 | 86.35 ± 13.62 |  | 40.35 ± 10.78 |  |
| **Inheritance patterns** |  |  | .51 |  | .17 |
| Autosomal dominant | 70 | 83.87 ± 12.89 |  | 38.57 ± 11.23 |  |
| Autosomal recessive | 33 | 85.48 ± 13.18 |  | 38.00 ± 12.18 |  |
| X-linked | 13 | 81.69 ± 13.89 |  | 43.31 ± 12.19 |  |
| Mitochondrial | 3 | 72.67 ± 11.68 |  | 51.67 ± 5.86 |  |
| Other | 40 | 84.92 ± 12.80 |  | 40.85 ± 11.88 |  |
| **Family history of genetic disease** |  |  | .91 |  | .99 |
| Yes | 22 | 84.36 ± 11.94 |  | 39.59 ± 10.10 |  |
| No | 137 | 84.04 ± 13.17 |  | 39.67 ± 11.97 |  |
| **Time since diagnosis** |  |  | .11 |  | .75 |
| <5 years | 102 | 83.58 ± 12.32 |  | 39.47 ± 11.57 |  |
| 5–10 years | 30 | 88.28 ± 13.70 |  | 38.91 ± 11.36 |  |
| 10–15 years | 13 | 80.25 ± 13.19 |  | 39.44 ± 13.63 |  |
| ≥15 years | 14 | 82.50 ± 14.18 |  | 42.50 ± 11.84 |  |
| **Disability registration** |  |  | .13 |  | **.004** |
| Registered | 36 | 80.69 ± 15.76 |  | 44.50 ± 12.14 |  |
| Not registered | 123 | 85.07 ± 11.92 |  | 38.24 ± 11.23 |  |
| **Treatment availability** |  |  | **.007** |  | **.001** |
| Available | 45 | 88.82 ± 11.75 |  | 35.33 ± 8.99 |  |
| Not available | 114 | 82.21 ± 13.00 |  | 41.37 ± 12.23 |  |
| **Copayment reduction registration** |  |  | .93 |  | .08 |
| Yes | 116 | 84.03 ± 13.27 |  | 40.61 ± 11.54 |  |
| No | 43 | 84.23 ± 12.26 |  | 37.09 ± 11.88 |  |

*Note.* Statistically significant p values (p < 0.05) are shown in bold. KCD, Korean Classification of Diseases; QoL, quality of life.

**Supplementary Table S3.** Caregiver quality of life and burden by genetic counseling experience (N = 159)

| **Scale** | **Genetic counseling experience** | **N** | **Mean ± SD** | ***P*-value** |
| --- | --- | --- | --- | --- |
| **Total QoL** | Yes | 50 | 84.70 ± 12.48 | .68 |
|  | No | 109 | 83.80 ± 13.23 |  |
| Family psychological health | Yes | 50 | 21.16 ± 2.74 | .63 |
|  | No | 109 | 20.92 ± 3.22 |  |
| Family burden | Yes | 50 | 17.22 ± 3.47 | .88 |
|  | No | 109 | 17.31 ± 4.08 |  |
| Community participation and support | Yes | 50 | 29.54 ± 6.18 | .69 |
|  | No | 109 | 29.97 ± 6.23 |  |
| Family openness | Yes | 50 | 8.40 ± 1.58 | **.013** |
|  | No | 109 | 7.72 ± 1.58 |  |
| Family cohesion | Yes | 50 | 8.38 ± 1.50 | .06 |
|  | No | 109 | 7.88 ± 1.68 |  |
| **Total burden** | Yes | 50 | 40.26 ± 11.57 | .66 |
|  | No | 109 | 39.39 ± 11.81 |  |
| Activity limitation | Yes | 50 | 10.06 ± 3.02 | .95 |
|  | No | 109 | 10.01 ± 3.11 |  |
| Social strain | Yes | 50 | 19.28 ± 5.81 | .40 |
|  | No | 109 | 18.62 ± 5.48 |  |
| Feelings of worry and guilt | Yes | 50 | 10.92 ± 3.64 | .90 |
|  | No | 109 | 10.83 ± 3.56 |  |

*Note.* Statistically significant p values (p < 0.05) are shown in bold. QoL, quality of life.
